# Oncological safety and preventive impact of nipple-sparing mastectomy in patients with *BRCA1/2* mutation: multicentre study of the Korea Robot-endoscopy Minimal Access Breast Surgery Study Group (KoREa-BSG)

**DOI:** 10.1093/bjsopen/zraf168

**Published:** 2026-02-17

**Authors:** Hong-Kyu Kim, Dong Seung Shin, Sung Yoon Jang, Soong June Bae, Eun Young Kim, Chihwan David Cha, Hyung Seok Park, Jeeyeon Lee, Jun-Hee Lee, Eun-Shin Lee, Jung Eun Choi, Soo Youn Bae, Hee-Chul Shin, Dongwon Kim, Moo Hyun Lee, Yong-Yeup Kim, Sang-Ah Han, Janghee Lee, Young Woo Chang, Junwon Min, Sanghwa Kim, Young-Joon Kang, Hee Jun Choi, Sae Byul Lee, Jai Min Ryu, Korea Robot-endoscopy Minimal Access Breast Surgery Study Group (KoREa-BSG), Joo Heung Kim, Beom Seok Ko, Ku Sang Kim, Young Jin Choi, Hye Yoon Lee, Sang Eun Nam, Zisun Kim, Jong Eun Lee, Eunhwa Park, Hyuk Jai Shin, Min Kyoon Kim, Seong Uk Kwon, Jeea Lee, Jee Ye Kim

**Affiliations:** Department of Surgery, Cancer Research Institute, Seoul National University College of Medicine, Seoul, Korea; Division of Breast Surgery, Department of Surgery, Samsung Medical Center, Sungkyunkwan University School of Medicine, Seoul, Korea; Division of Breast Surgery, Department of Surgery, Jeju National University Hospital, Jeju National University School of Medicine, Jeju, Korea; Department of Surgery, Gangnam Severance Hospital, Yonsei University College of Medicine, Seoul, Korea; Department of Surgery, Kangbuk Samsung Hospital, Sungkyunkwan University School of Medicine, Seoul, Korea; Department of Surgery, Hanyang University Medical Center, Hanyang University College of Medicine, Seoul, Korea; Division of Breast Surgery, Department of Surgery, Yonsei University College of Medicine, Seoul, Korea; Department of Surgery, School of Medicine, Kyungpook National University, Kyungpook National University Chilgok Hospital, Daegu, Korea; Department of Surgery, Soonchunhyang University College of Medicine, Soonchunhyang University Hospital, Seoul, Korea; Division of Breast and Endocrine Surgery, Department of Surgery, Korea University Anam Hospital, Korea University College of Medicine, Seoul, Korea; Department of Surgery, Yeungnam University College of Medicine, Daegu, Korea; Department of Surgery, Seoul St. Mary's Hospital, The Catholic University of Korea, Seoul, Korea; Department of Surgery, Seoul National University Bundang Hospital, Seoul National University College of Medicine, Seongnam, Korea; Department of Surgery, Daerim St. Mary's Hospital, Seoul, Korea; Department of Surgery, Keimyung University School of Medicine, Daegu, Korea; Division of Breast and Endocrine Surgery, Department of Surgery, Korea University Guro Hospital, Korea University College of Medicine, Seoul, Korea; Department of Surgery, Kyung Hee University Hospital at Gangdong, School of Medicine, Kyung Hee University, Seoul, Korea; Department of Surgery, Ewha Womans University Mokdong Hospital, Ewha Womans University College of Medicine, Seoul, Korea; Department of Medicine, Yonsei University College of Medicine, Seoul, Korea; Division of Breast and Endocrine Surgery, Department of Surgery, Korea University Ansan Hospital, Korea University College of Medicine, Seoul, Korea; Department of Surgery, Dankook University College of Medicine, Cheonan, Korea; Department of Breast and Endocrine Surgery, Hallym University Sacred Heart Hospital, Hallym University, Anyang, Korea; Department of Surgery, Incheon St. Mary’s Hospital, The Catholic University of Korea, Incheon, Korea; Department of Surgery, Samsung Changwon Hospital, Sungkyunkwan University School of Medicine, Changwon, Korea; Department of Surgery, University of Ulsan College of Medicine, Asan Medical Center, Seoul, Korea; Division of Breast Surgery, Department of Surgery, Samsung Medical Center, Sungkyunkwan University School of Medicine, Seoul, Korea

**Keywords:** breast cancer, contralateral breast cancer, risk-reducing surgery

## Abstract

**Background:**

Nipple-sparing mastectomy (NSM) is a surgical option offering both oncological safety and cosmetic benefits. However, the oncological safety of NSM in carriers of *BRCA1/2* pathogenic variants/likely pathogenic variants (PV/LPV) with breast cancer and the role of risk-reducing mastectomy remain underexplored, especially in Asian populations. This study evaluated the safety and effectiveness of NSM in *BRCA1/2* PV/LPV carriers and assessed the preventive impact of contralateral risk-reducing NSM (RRNSM) on cancer incidence.

**Methods:**

This multicentre retrospective study included women aged 20–80 years who underwent NSM for therapeutic or risk-reducing purposes and received germline *BRCA1/2* tests between May 2006 and June 2022 across 19 institutions in Korea. Patients with distant metastasis at diagnosis were excluded. Information on demographics, the clinical characteristics of patients and tumours, surgical details, and follow-up outcomes was collected from a review the medical records of each participating institution. The primary outcome was the oncological safety of NSM, assessed by comparing ipsilateral local recurrence rates between patients with and without *BRCA1/2* PV/LPV. The secondary outcome was cancer incidence in patients who underwent contralateral RRNSM *versus* those who did not.

**Results:**

In all, 787 women underwent 906 NSMs, with a median (interquartile range) follow-up of 59.3 (44.0–82.8) months. Among the participants, 186 (23.6%) were *BRCA1/2* PV/LPV carriers. Ipsilateral local recurrence rates were comparable between *BRCA1/2* PV/LPV carriers and non-carriers (6.4 *versus* 7.4%, respectively). The 5-year local recurrence-free survival rates did not differ significantly between *BRCA1/2* PV/LPV carriers and non-carriers (92.2% *versus* 93.2%, respectively; *P* = 0.87). Contralateral breast cancer occurred in 4.5% of patients with *BRCA1/2* PV/LPV who did not undergo contralateral RRNSM, whereas no cases of contralateral breast cancer were reported among patients who underwent RRNSM regardless of *BRCA1/2* status.

**Conclusions:**

This study highlights NSM as a safe and effective surgical option for *BRCA1/2* PV/LPV carriers with breast cancer, as well as a risk-reducing strategy. Further prospective studies are needed to confirm these findings and evaluate long-term outcomes.

## Teaser

This multicentre study evaluated the oncological safety and preventive potential of nipple-sparing mastectomy (NSM) in carriers of *BRCA1/2* mutations. NSM had comparable local recurrence rates to traditional mastectomy and a reduced incidence of contralateral breast cancer, supporting its role as a viable surgical option for high-risk breast cancer patients.

## Introduction

Nipple-sparing mastectomy (NSM) with reconstruction preserves the nipple–areola complex, potentially improving patient satisfaction with body image compared with skin-sparing mastectomy (SSM). A recent systematic review^1^ further support generally positive psychosocial outcomes associated with risk-reducing mastectomy (RRM), although patient-reported experiences regarding aesthetic results and body image vary, emphasizing the need for the cautious interpretation of these findings. NSM, by keeping the nipple–areola complex while removing the underlying breast tissue, marks a significant advance in breast cancer management, providing both oncological safety and cosmetic benefits^2–5^. Several studies^6–8^ have demonstrated that NSM achieves comparable oncological outcomes to SSM or conventional total mastectomy, with no significant difference in recurrence rates and overall survival. Furthermore, NSM is increasingly being used for patients undergoing neoadjuvant chemotherapy and those with locally advanced breast cancer, particularly when immediate reconstruction is planned^9,10^.

Although many studies have been reported on the oncological outcomes of NSM, there is very limited data on its safety and effectiveness in patients with pathogenic variants (PV) or likely pathogenic variants (LPV) of the *BRCA1/2* genes. Given that *BRCA1/2*-related breast cancers are often diagnosed at a younger age and tend to have more aggressive tumour biology, evaluating the safety of NSM in this subgroup is crucial^11,12^. In particular, there have been no studies investigating the oncological outcomes of NSM in patients with *BRCA1/2*-related breast cancer in an Asian population.

In *BRCA1/2* PV/LPV carriers diagnosed with breast cancer, the risk of contralateral breast cancer (CBC) events increases progressively over time, with cumulative incidence rates reaching 23% for *BRCA1* and 17% for *BRCA2* mutation patients at 10 years after the initial diagnosis^13^. These findings highlight the critical need for personalized surveillance plans and risk-reducing strategies, including surgical interventions with NSM, to address cancer risks in this high-risk population. Moreover, the role of NSM as a preventive measure in unaffected carriers of *BRCA1/2* PV/LPV also needs thorough investigation. These individuals frequently struggle with the decision whether to undergo preventive surgeries to reduce their risk of cancer. It is therefore essential to determine whether NSM can serve as an effective risk-reducing strategy for these high-risk individuals, while maintaining as high a quality of life and satisfaction as possible.

The aims of this study were to assess the oncological outcomes of NSM for patients with breast cancer with *BRCA1/2* PV/LPV and the contralateral preventive effectiveness of NSM in these high-risk patients.

## Methods

### Study population and data collection

A multicentre retrospective study was conducted by the Korea Robot-endoscopic Minimal Access Breast Surgery Study Group (KoREa-BSG), a surgical study group within the Korean Breast Cancer Society. As part of this study, women who underwent NSM and received germline *BRCA1/2* tests between May 2006 and June 2022 were retrospectively identified across 19 institutions in Korea. The study included women aged 20–80 years who underwent NSM, regardless of whether the surgery was for therapeutic or risk-reducing purposes. Individuals who were diagnosed with distant metastasis at the initial presentation were excluded from the study.

Information was collected on demographic data, the clinical characteristics of patients and tumours, surgical details, and follow-up outcomes from a review of the medical records of each participating institution. Pathological and clinical staging in this study were determined according to the 8th edition of the American Joint Committee on Cancer classification^14^. The results of germline *BRCA1/2* testing were categorized into five groups: *BRCA1* PV/LPV, *BRCA2* PV/LPV, variants of uncertain significance, negative, and equivocal. Among these, individuals with variants of uncertain significance, negative, or equivocal results were collectively classified as *BRCA* negative for the purposes of analysis. According to the American College of Medical Genetics and Genomics guidelines^15^, the term ‘likely pathogenic’ is used for variants with greater than 90% certainty of being disease causing even though they do not yet meet the highest level of evidence required for definitive pathogenic classification. Therefore, LPVs are routinely categorized alongside PVs for clinical analyses and decision-making.

Locoregional recurrence was defined as any recurrence occurring in the ipsilateral chest wall, skin, subcutaneous tissue, or pectoralis muscle, which was categorized as local recurrence, or within the ipsilateral axillary, supraclavicular, internal mammary, or infraclavicular lymph nodes, which was classified as regional recurrence. Distant metastasis was defined as any recurrence occurring outside the regions classified under local recurrence or regional recurrence. Data on recurrence events were extracted from electronic medical records, whereas survival information was sourced from both institutional electronic records and the Korean National Statistical Office database.

In this study, postoperative surveillance generally followed the clinical guidelines of the Korean Breast Cancer Society. According to these guidelines, physical examination and history taking are performed during outpatient clinic visits every 6 months for the first 3 years, every 6–12 months for the next 2 years, and annually thereafter. Imaging of the breast, including mammography or breast ultrasound, is performed every 6–12 months, with breast magnetic resonance imaging added if clinically indicated. Systemic imaging to detect distant metastasis was performed every 6–12 months as needed, depending on the patient’s disease stage.

The primary outcome in this study was the oncological safety of NSM, assessed by comparing ipsilateral local recurrence rates between patients with and without *BRCA1/2* PV/LPV. The secondary outcome was cancer incidence in patients who underwent contralateral risk-reducing NSM (RRNSM) *versus* those who did not.

This study was conducted in accordance with the ethical principles of the Declaration of Helsinki. The study was approved by the Institutional Review Board of Samsung Medical Center (Approval no. 2022-01-195), as well as the institutional review boards of each participating institution. The requirement for informed consent was waived because of the retrospective nature of the study.

### Statistical analysis

Variables were compared between groups using the χ^2^ test or Fisher's exact test for categorical variables and the *t* test or Wilcoxon rank-sum test for continuous variables. Kaplan–Meier curves were generated for local recurrence-free survival, regional recurrence-free survival, distant metastasis-free survival, and overall survival with the corresponding outcomes of log-rank tests. All statistical analyses were conducted using SPSS^®^ version 27 (IBM, Armonk, NY, USA) and R version 4.2.2 (Foundation for Statistical Computing, Vienna, Austria). All statistical tests were two-sided, with *P* < 0.050 considered statistically significant, and results are reported with 95% confidence intervals (c.i.).

## Results

### Patient demographics

In all, 787 women (906 NSMs) were included in the study. There were two unaffected individuals with *BRCA1/2* PV/LPV who underwent bilateral RRNSMs. The median follow-up period was 59.3 (interquartile range 44.0–82.8) months, and the median age of the cohort was 39 (range 23–79) years. Sentinel lymph node biopsy (SLNB) was performed in 784 NSMs (86.5%, 784 of 906) and 198 patients (25.2%, 198 of 785) received neoadjuvant chemotherapy.

Among the 746 patients with unilateral breast cancer, 659 underwent unilateral NSM only, 78 underwent unilateral NSM with contralateral RRNSM, and nine underwent unilateral total mastectomy with contralateral RRNSM, with 112, 60, and six *BRCA1/2* PV/LPV carriers in each group, respectively. In addition, the cohort included 39 patients with bilateral breast cancer who underwent bilateral NSM, of whom six were *BRCA1/2* PV/LPV carriers and 33 were non-carriers (*Table 1*).

**Table 1:** Numbers of patients with unilateral and bilateral breast cancer, procedures, and outcomes (n = 787)

	All patients (*n*)	*BRCA1/2* negative (*n* = 601)	*BRCA1/2* PV/LPV (*n* = 186)
**Unilateral breast cancer**			
Unilateral NSM (Group A)	659	547 (91.0%)	112 (60.2%)
Unilateral TM + contralateral RRNSM^A^ (Group B)	9	3 (0.5%)	6 (3.2%)
Unilateral NSM + contralateral RRNSM^A^ (Group C)	78	18 (3.0%)	60 (32.3%)
**Bilateral breast cancer**			
Bilateral NSM	39	33 (5.5%)	6 (3.2%)
**Bilateral RRNSM** ^B^	2	0 (0.0%)	2 (1.1%)
^A^Results of contralateral RRNSM (*n*)	87	21	66
Benign tumour after contralateral RRNSM	85	20	65
Incidental cancer after contralateral RRNSM	2	1	1
^B^Results of bilateral RRNSM (*n*)	2	0	2
Benign tumour after bilateral RRNSM	2	0	2
Incidental cancer after bilateral RRNSM	0	0	0

Values are *n* (%) unless otherwise stated. PV/LPV, pathogenic variant/likely pathogenic variant; NSM, nipple-sparing mastectomy; TM, total mastectomy; RRNSM, risk-reducing nipple-sparing mastectomy.

Among the 785 patients with breast cancer, excluding the two unaffected individuals with *BRCA1/2* PV/LPV who underwent bilateral RRNSMs, there were differences in baseline characteristics between the *BRCA1/2* PV/LPV and *BRCA1/2*-negative groups (*Table 2*). The number of patients aged ≤ 40 years was significantly higher in the *BRCA1/2* PV/LPV than *BRCA1/2*-negative group (69.6 *versus* 59.7%, respectively; *P* =0.039). In addition, the *BRCA1/2* PV/LPV group had a higher proportion of patients who were hormone receptor-negative (32.6 *versus* 17.0%; *P* < 0.001) and a higher prevalence of triple-negative breast cancer (27.2 *versus* 7.5%, *P* < 0.001). The proportion of patients with a Ki-67 index ≥ 20% was also significantly higher in the *BRCA1/2* PV/LPV than *BRCA1/2*-negative group (49.5 *versus* 34.3%; *P* < 0.001), suggesting a more aggressive tumour biology in the *BRCA1/2* PV/LPV cohort. Regarding axillary surgery, the distribution differed significantly between the two groups (*P* = 0.001), with the *BRCA1/2* PV/LPV group having a higher proportion of patients undergoing axillary lymph node dissection following SLNB (21.2 *versus* 18.6%), as well as a higher overall rate of axillary lymph node dissection (25.5 *versus* 22.8%).

**Table 2:** Baseline characteristics of *BRCA1/2*-negative and *BRCA1/2* PV/LPV groups

Variables	*BRCA1/2* negative (*n* = 601)	*BRCA1/2* PV/LPV (*n* = 184)*	*P*†
Age at operation (years), median (i.q.r.)	39 (35–46)	37 (34–42)	0.002
Follow-up duration (months), median (i.q.r.)	59.6 (44.8–83.8)	55.4 (42.3–76.6)	0.092
**pT**			0.024
pTis	134 (22.3%)	40 (21.7%)	
pT1	290 (48.3%)	102 (55.4%)	
pT2	143 (23.8%)	34 (18.5%)	
pT3	26 (4.3%)	2 (1.1%)	
pT4	2 (0.3%)	0 (0.0%)	
Unknown	6 (1.0%)	6 (3.3%)	
**pN**			0.216
pN0	435 (72.4%)	124 (67.4%)	
pN1	125 (20.8%)	44 (23.9%)	
pN2	23 (3.8%)	6 (3.3%)	
pN3	12 (2.0%)	4 (2.2%)	
Unknown	6 (1.0%)	6 (3.3%)	
**Axillary surgery**			0.001
No	9 (1.5%)	13 (7.1%)	
SLNB	455 (75.7%)	124 (67.4%)	
SLNB followed by ALND	122 (18.6%)	39 (21.2%)	
ALND	25 (4.2%)	8 (4.3%)	
**Hormone receptor**			<0.001
Positive	473 (78.7%)	103 (56.0%)	
Negative	102 (17.0%)	60 (32.6%)	
Unknown	26 (4.3%)	21 (11.4%)	
**HER2 status**			0.003
Positive	128 (21.3%)	22 (12.0%)	
Negative	383 (63.7%)	120 (65.2%)	
Unknown	90 (15.0%)	42 (22.8%)	
**Molecular subtype**			<0.001
HR+/HER2−	338 (56.2%)	70 (38.0%)	
HR+/HER2+	80 (13.3%)	15 (8.2%)	
HR−/HER2+	48 (8.0%)	7 (3.8%)	
HR−/HER2−	45 (7.5%)	50 (27.2%)	
Unknown	90 (15.0%)	42 (22.8%)	
**Ki-67**			<0.001
< 20%	390 (64.9%)	87 (47.3%)	
≥ 20%	206 (34.3%)	91 (49.5%)	
Unknown	5 (0.8%)	6 (3.3%)	
**Histological grade**			<0.001
Grade 1	57 (9.5%)	5 (2.7%)	
Grade 2	304 (50.6%)	74 (40.2%)	
Grade 3	121 (20.1%)	63 (34.2%)	
Unknown	119 (19.8%)	42 (22.8%)	

Values are *n* (%) unless otherwise stated. *Two unaffected individuals who underwent bilateral risk-reducing nipple-sparing mastectomy were excluded from this comparison. PV/LPV, pathogenic variant/likely pathogenic variant; i.q.r., interquartile range; pT, pN, pathological T and N categories, respectively; Tis, TNM *in situ*; SLNB, sentinel lymph node biopsy; ALND, axillary lymph node dissection; HER2, human epidermal growth factor receptor 2; HR, hormone receptor. †*P* values were calculated using the Wilcoxon rank-sum test for continuous variables and the *χ*^2^ test or Fisher’s exact test, as appropriate, for categorical variables.

### Results of *BRCA1/2* genetic testing

In this retrospective study, *BRCA1* and *BRCA2* PV/LPV were identified in 85 (10.8%) and 101 (12.8%) individuals, respectively (*Table S1*). Most individuals were confirmed negative for *BRCA1/2* mutations (522 patients, 66.3%). Variants of unknown significance and equivocal results were identified in 76 (9.7%) and 3 (0.4%) patients, respectively.

### Ipsilateral local recurrence (oncological outcomes of NSM according to *BRCA1/2* mutation status)

**Fig. 1:**
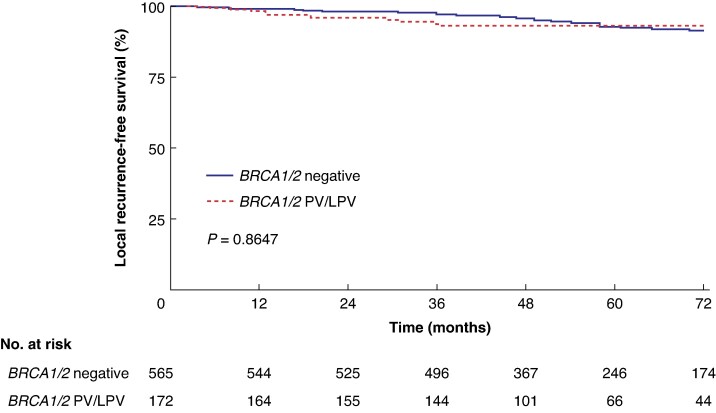
Ipsilateral local recurrence-free survival in patients with unilateral nipple-sparing mastectomy according to *BRCA1/2* status This analysis evaluated the impact of nipple-sparing mastectomy on ipsilateral local recurrence-free survival in 737 patients with unilateral breast cancer (after excluding nine patients who underwent unilateral total mastectomy). The *P* value was calculated using a log-rank test. PV/LPV, pathogenic variant/likely pathogenic variant.

To evaluate the oncological safety of NSM in *BRCA1/2* PV/LPV carriers, ipsilateral local recurrence rates were analysed according to *BRCA1/2* mutation status. For this analysis, patients with unilateral breast cancer who underwent ipsilateral NSM were selected (*Table 1*, groups A and C). No significant difference in local recurrence was observed between the 172 patients with *BRCA1/2* PV/LPV and the 565 patients without *BRCA1/2* PV/LPV. Specifically, 11 local recurrence events (6.4%, 11 of 172) occurred in the *BRCA1/2* PV/LPV group, compared with 42 events (7.4%, 42 of 565) in the *BRCA1/2*-negative group. There was no significant difference in local recurrence-free survival between the two groups (log-rank *P* = 0.865; *Fig. 1*). Furthermore, no significant differences were noted in regional recurrence-free survival, distant metastasis-free survival, or overall survival (log-rank *P* = 0.707, *P* = 0.156, and *P* = 0.373, respectively; *Fig. S1*).

### CBC occurrence (impact of RRNSM on CBC incidence)

**Fig. 2:**
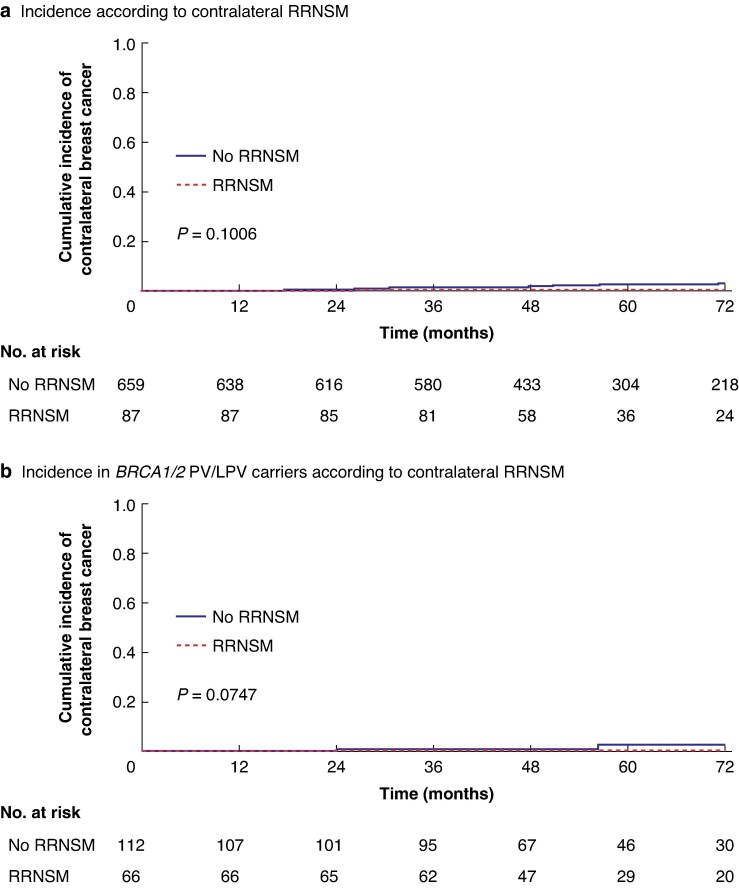
Cumulative incidence of contralateral breast cancer according to contralateral RRNSM status **a** Cumulative incidence of contralateral breast cancer in patients with unilateral breast cancer who did not (659 patients) or did (87 patients) undergo contralateral RRNSM. **b** Cumulative incidence of contralateral breast cancer in patients identified as *BRCA1/2* PV/LPV carriers who did not (112 patients) or did (66 patients) undergo contralateral RRNSM. The *P* values were calculated using log-rank tests. RRNSM, risk-reducing nipple-sparing mastectomy; PV/LPV, pathogenic variant/likely pathogenic variant.

To evaluate the impact of RRNSM on CBC, the incidence of CBC was analysed according to *BRCA1/2* mutation status. For this analysis, patients with unilateral breast cancer were selected and classified into two groups according to whether or not they underwent contralateral RRNSM (*Table 1*, group A *versus* groups B + C). Among the 659 patients with unilateral NSM who did not undergo RRNSM, CBC occurred in 22 patients (3.3%), whereas no cases were reported among the 87 patients who underwent RRNSM. There was no significant difference in cumulative CBC occurrence between the two groups (log-rank *P* = 0.101; *Fig. 2a*). In the subgroup of 178 patients with *BRCA1/2* PV/LPV, CBC occurred in five of 112 patients (4.5%) who did not undergo RRNSM, whereas no cases were observed among the 66 patients who underwent RRNSM. Kaplan–Meier survival analysis in this subgroup also demonstrated no statistically significant difference in cumulative CBC occurrence between the two groups (log-rank *P* = 0.075; *Fig. 2b*).

### Pathological results of RRNSM

Among the patients with unilateral breast cancer, 87 underwent contralateral RRNSM for risk reduction, including 66 with *BRCA1/2* PV/LPV and 21 without. Among these 87 patients with contralateral RRNSM, benign tumours were found in 85 (65 with *BRCA1/2* PV/LPV and 20 without), whereas incidental ductal carcinoma *in situ* was identified in two patients, one with *BRCA1/2* PV/LPV and one without. In addition, two individuals underwent bilateral RRNSMs, both of whom were *BRCA1/2* PV/LPV carriers. The final pathological report of both breasts in these carriers revealed only benign tumours, with no incidental cancer identified.

## Discussion

The results of this study demonstrate that NSM is a viable and safe surgical option for patients with breast cancer with *BRCA1/2* PV/LPV, showing no significant difference in ipsilateral local recurrence rates between *BRCA1/2* PV/LPV carriers and non-carriers. Furthermore, the risk-reducing potential of contralateral RRNSM was evident, because no cases of CBC were observed among individuals with *BRCA1/2* PV/LPV who underwent the procedure, although no statistically significant difference was observed. The findings of the present multicentre, retrospective study provide valuable insights into the oncological safety and preventive benefits of NSM in patients with *BRCA1/2* PV/LPV.

One of the primary concerns regarding NSM in *BRCA1/2* PV/LPV carriers has been the potential risk of local recurrence due to the preservation of the nipple–areola complex. However, recent studies^4,16–20^ have reported on the oncological safety of NSM as a risk-reducing mastectomy option for *BRCA1/2* PV/LPV carriers (*Table 3*). Jakub *et al*.^18^ analysed 548 cases of prophylactic NSM in 346 *BRCA* mutation carriers across multiple institutions, reporting no new breast cancer events during a median follow-up of 34 months. Valero *et al*.^21^ evaluated 384 bilateral prophylactic NSMs in 192 patients, including 117 *BRCA1/2* mutation carriers, and found no new breast cancer cases during a median follow-up of 36.8 months. Similarly, Webster *et al*.^4^ conducted a long-term analysis of 114 cases of therapeutic NSM in 105 *BRCA1/2* mutation carriers over a median follow-up of 70 months, reporting a locoregional recurrence rate of only 2.6%. To date, there is a notable lack of research on the safety of NSM or RRNSM in *BRCA1/2* PV/LPV carriers within the Asian population^22^. The present study bridges this gap as the first and largest multicentre cohort in Asia to evaluate oncological outcomes and the preventive potential of NSM in this high-risk group. The findings of no significant difference in ipsilateral local recurrence rates between *BRCA1/2* PV/LPV carriers and non-carriers align with those of previous studies, suggesting that NSM offers comparable oncological safety to conventional mastectomy techniques, even in high-risk populations.

**Table 3:** Comparative summary of studies evaluating nipple-sparing mastectomy in carriers of BRCA mutations

Study	No. institutions	Country	Sample size (*n*)	No. of NSM procedures	No. of *BRCA* carriers	Objective	Median follow-up duration (months)	Key findings
Stanek *et al*.^20^ (2022)	Single	Czech Republic	105	210	105	Risk-reducing effectiveness of NSM	50	Cancer rates: NSM group, 0%; surveillance group, 8.6% cancer
Garstka *et al*.^19^ (2021)	Single	Germany	307	607	307	Ipsilateral recurrence rates in NSM	42	No new cancers in prophylactic NSM Therapeutic NSM had low recurrence rates
Manning *et al*.^16^ (2015)	Single	USA	89	177	89	Indications and outcomes in *BRCA* mutation carriers	28	Therapeutic NSM: no recurrence Prophylactic NSM: 6% DCIS
Webster *et al*.^4^ (2023)	Single	USA	105	114	105	Long-term oncological safety of NSM	70	2.6% locoregional recurrence No nipple recurrence 96% survival
Valero *et al*.^21^ (2020)	Single	USA	192	384	117	Outcomes of bilateral prophylactic NSM	36.8	0% new breast cancer diagnoses Low complication rates
Jakub *et al*.^18^ (2017)	Multiple	USA	346	548	346	Oncological safety of prophylactic NSM for *BRCA* mutation carriers	34	No new breast cancers occurred Significantly fewer events compared to expected without NSM

NSM, nipple-sparing mastectomy; DCIS, ductal carcinoma *in situ*.

Survival analyses in the present cohort revealed an interesting trend. Patients with *BRCA1/2* PV/LPV experienced more frequent events early on, but the survival curves subsequently crossed during follow-up. This pattern could be explained by the more aggressive tumour biology and higher prevalence of the triple-negative subtype in the *BRCA1/2* PV/LPV group, leading to earlier recurrence. Over time, however, the curves converge, suggesting that the initial adverse impact may diminish. It is also important to note that the risk of local recurrence could be underestimated, because patients who developed distant metastasis or died were censored and therefore not recorded for subsequent local events. These findings underscore the complexity of interpreting recurrence patterns in genetically high-risk populations.

A previous systematic review and meta-analysis^23^ and a study^24^ have reported that, among patients undergoing breast-conserving surgery, *BRCA1/2* mutation carriers have a higher risk of ipsilateral breast tumour recurrence than non-carriers^23,24^. However, a recent study by Moshe *et al*.^25^ analysing patients who underwent SSM or NSM reported that *BRCA1/2* mutation status did not affect the local recurrence rate. This may suggest that mastectomy has a more pronounced impact in reducing ipsilateral breast tumour recurrence, although further studies are warranted to confirm this. Regarding occult malignancy, studies conducted in various countries^26–28^ have shown that the incidence of occult malignancy in the contralateral breast undergoing RRM in *BRCA1/2* mutation carriers ranges from 2.4 to 11.3%. In a recent study from a single institution in Korea^29^, among 320 breast cancer patients who underwent contralateral RRM, seven cases (2.2%) of occult malignancy were identified in *BRCA1/2* mutation carriers. Similarly, the present study showed a low rate of incidental malignancy; however, given the small number of cases and the inclusion of only patients who underwent NSM, the generalizability of this finding is limited. In line with this, Thompson *et al*.^30^ reported that *BRCA1/2* mutation status was not a statistically significant factor influencing the risk of occult malignancy in patients undergoing contralateral RRM. Further studies with larger cohorts and prospective randomized designs are needed to better understand the risk of local recurrence and incidental malignancy in *BRCA1/2* mutation carriers.

Previous studies^31,32^ have reported that RRM reduces the risk of breast cancer; however, its overall survival benefit remains controversial. According to the most recent study by Blondeaux *et al*.^33^, RRM or risk-reducing salpingo-oophorectomy may provide overall survival gain in patients with *BRCA1* mutations identified before the age of 40 years. In addition, long-term follow-up results for patients with triple-negative breast cancer with confirmed *BRCA1/2* mutations showed that 82.8% of CBC events were of the triple-negative breast cancer subtype, and 62.5% of these patients received systemic chemotherapy again^34^. Therefore, from a long-term perspective, patients may opt for RRM considering the future risk of CBC and their personal preferences. Compared with conventional mastectomy or SSM, both of which do not preserve the nipple–areolar complex, NSM has been associated with greater patient satisfaction, particularly in terms of sexual and psychosocial wellbeing^35^. Therefore, choosing NSM with immediate reconstruction can offer the benefits of risk reduction and potentially favourable oncological outcomes, while also aiming to enhance overall patient satisfaction. In particular, the cohort in the present study had a relatively young median age of 39 years. Given the long life expectancy in this population, multiple factors need to be considered, including oncological outcomes of surgery, cosmetic benefits, quality of life, fertility preservation, and family planning. In this context, a multidisciplinary approach and shared decision-making that focuses on tailoring treatment strategies to patient preferences and clinical factors should be further highlighted.

With recent advances in minimal access breast surgery, robot-assisted NSM (RANSM) has emerged as a new option for NSM. RANSM has demonstrated favourable oncological outcomes and superior cosmetic results in women with early breast cancer or *BRCA* mutations, with multiple studies^36–39^ reporting no significant difference in complication rates compared with conventional NSM while maintaining postoperative quality of life. Several ongoing multicentre trials, including randomized studies in Korea (NCT05490433) and the USA (NCT05720039), are currently evaluating the safety, oncological outcomes, and patient satisfaction associated with RANSM^40^. The findings from these studies are expected to provide valuable insights into whether RANSM can serve as an alternative to conventional NSM for high-risk patients, and whether it could offer superior oncological outcomes and patient satisfaction as a risk-reducing technique.

Although this study provides important evidence supporting the oncological safety of NSM in patients with *BRCA1/2* PV/LPV, several limitations should be noted. First, the retrospective design may have introduced selection bias, and the relatively short median follow-up period of 60 months may not fully capture long-term recurrence patterns. However, although several studies have assessed the oncological safety of NSM in *BRCA* mutation carriers, most have been limited to single-centre cohorts, and multicentre data remain limited. In particular, evidence from Asian populations is notably lacking. This appears to be the first multicentre retrospective analysis conducted in an Asian population that includes patients who underwent NSM with known *BRCA1/2* PV/LPV status. By analysing oncological outcomes and the preventive effect of NSM on contralateral breast cancer in this genetically high-risk population, the present study helps fill an important gap in the literature and provides clinically meaningful insights into the surgical management of *BRCA* mutation carriers.

Second, although multivariable analysis could have improved the validity of recurrence and survival outcomes by adjusting for confounding factors, it was not feasible due to incomplete and inconsistent reporting of key variables such as adjuvant treatment details, Ki-67 index, and other potential prognostic factors across participating institutions. Moreover, the influence of unmeasured or unknown confounding factors could not be excluded due to the retrospective design of the study.

Third, data on bilateral salpingo-oophorectomy status, a known protective factor for breast cancer development, contralateral events, and recurrence, were not comprehensively collected. Therefore, the potential influence of bilateral salpingo-oophorectomy could not be evaluated in this study.

Future prospective, multicentre studies with standardized data collection and longer follow-up are warranted to confirm these findings and provide further insights into the preventive and therapeutic impacts of NSM in high-risk populations.

In this retrospective study, NSM was found to be a safe and effective surgical option for patients with breast cancer with *BRCA1/2* PV/LPV. It was associated with a low risk of ipsilateral local recurrence and a reduced incidence of CBC in affected *BRCA1/2* PV/LPV carriers. These findings support the consideration of NSM as a potential surgical approach in the management of high-risk patients and may contribute to improved quality of life. Further research is needed to validate these findings and to explore the long-term impact of NSM in this population.

## Collaborators

The members of the KoREa-BSG are: Joo Heung Kim (Department of Surgery, Yongin Severance Hospital, Yonsei University College of Medicine, Yongin, Korea); Beom Seok Ko (Department of Surgery, Asan Medical Center, University of Ulsan College of Medicine, Seoul, Korea); Ku Sang Kim (Department of Surgery, Kosin University College of Medicine, Gospel Hospital, Busan, Korea); Young Jin Choi (Department of Surgery, Chungbuk National University Hospital, Cheongju, Korea); Hye Yoon Lee (Department of Surgery, Korea University Ansan Hospital, Ansan, Korea); Sang Eun Nam (Department of Surgery, Konkuk University School of Medicine, Seoul, Korea); Zisun Kim (Department of Surgery, Soonchunhyang University Bucheon Hospital, Bucheon, Korea); Jong Eun Lee (Department of Surgery, Soonchunhyang University Cheonan Hospital, Cheonan, Korea); Eunhwa Park (Department of Surgery, Dong-A University Hospital, Dong-A University College of Medicine, Busan, Korea); Hyuk Jai Shin (Department of Surgery, Myongji Hospital, Hanyang University Medical Center, Goyang, Korea); Min Kyoon Kim (Department of Surgery, Chung-Ang University Hospital, Seoul, Korea); Seong Uk Kwon (Department of Surgery, Konyang University Hospital, Daejeon, Korea); Jeea Lee (Department of Surgery, Uijeongbu Eulji Medical Center, Eulji University, Uijeongbu, Korea); and Jee Ye Kim (Department of Surgery, Yonsei University College of Medicine, Seoul, Korea).

## Supplementary Material

zraf168_Supplementary_Data

## Data Availability

J.M.R. and S.B.L. had full access to all of the data in the study and take responsibility for the integrity of the data and the accuracy of the data analysis. The data that support the findings of this study are available on request from the corresponding authors, after proper revision of the data transfer agreement of the institutions and if ultimately allowed by ethic committees.
