# Supplementary material for: Oncological safety and preventive impact of nipple-sparing mastectomy in patients with BRCA1/2 mutation: multicentre study of the Korea Robot-endoscopy Minimal Access Breast Surgery Study Group (KoREa-BSG)
Source: BJS Open. 2026 Feb 17;10(1):zraf168. doi: 10.1093/bjsopen/zraf168 (PMC12911035; doi:10.1093/bjsopen/zraf168)
Supplement: zraf168_Supplementary_Data [file zraf168_supplementary_data.docx]

**Oncologic Safety and Preventive Impact of Nipple-Sparing Mastectomy in Patients with *BRCA1/2* Mutation: Multicentre Retrospective Study of the Korea Robot-Endoscopy Minimal Access Breast Surgery Study Group (KoREa-BSG)**

Hong-Kyu Kim^1,*^, MD, PhD; Dong Seung Shin^2,*,†^, MD; Sung Yoon Jang^3^, MD; Soong June Bae^4^, MD; Eun Young Kim^5^, MD, PhD; Chihwan David Cha^6^, MD, PhD; Hyung Seok Park^7^, MD, PhD; Jeeyeon Lee^8^, MD, PhD; Jun-Hee Lee^9^, MD, PhD; Eun-Shin Lee^10^, MD; Jung Eun Choi^11^, MD, PhD; Soo Youn Bae^12^, MD, PhD; Hee-Chul Shin^13^, MD, PhD; Dongwon Kim^14^, MD; Moo Hyun Lee^15^, MD; Yong-Yeup Kim^16^, MD; Sang-Ah Han^17^, MD, PhD; Janghee Lee^18,19^, MD; Young Woo Chang^20^, MD; Junwon Min^21^, MD; Sanghwa Kim^22^, MD; Young-Joon Kang^23^ MD, PhD; Hee Jun Choi^24^, MD, PhD; Sae Byul Lee^25^, MD, PhD; Jai Min Ryu^2^, MD, PhD; and Korea Robot-Endoscopy minimal access Breast surgery Study Group (KorREa-BSG)

^1^Department of Surgery, Cancer Research Institute, Seoul National University College of Medicine, Seoul, Korea

^2^Division of Breast Surgery, Department of Surgery, Samsung Medical Center, Sungkyunkwan University School of Medicine, Seoul, Korea

^3^Division of Breast Surgery, Department of Surgery, Jeju National University Hospital, Jeju National University School of Medicine, Jeju, Korea

^4^Department of Surgery, Gangnam Severance Hospital, Yonsei University College of Medicine, Seoul, Korea

^5^Department of Surgery, Kangbuk Samsung Hospital, Sungkyunkwan University School of Medicine, Seoul, Korea

^6^Department of Surgery, Hanyang University Medical Center, Hanyang University College of Medicine, Seoul, Korea

^7^Division of Breast Surgery, Department of Surgery, Yonsei University College of Medicine, Seoul, Korea

^8^Department of Surgery, School of Medicine, Kyungpook National University, Kyungpook National University Chilgok Hospital, Daegu, Korea

^9^Department of Surgery, Soonchunhyang University College of Medicine, Soonchunhyang University Hospital, Seoul, Korea

^10^Division of Breast and Endocrine Surgery, Department of Surgery, Korea University Anam Hospital, Korea University College of Medicine, Seoul, Korea

^11^Department of Surgery, Yeungnam University College of Medicine, Daegu, Korea

^12^Department of Surgery, Seoul St. Mary's Hospital, The Catholic University of Korea, Seoul, Korea

^13^Department of Surgery, Seoul National University Bundang Hospital, Seoul National University College of Medicine, Seongnam, Korea

^14^Department of Surgery, Daerim St. Mary's Hospital, Seoul, Korea

^15^Department of Surgery, Keimyung University School of Medicine, Daegu, Korea

^16^Division of Breast and Endocrine Surgery, Department of Surgery, Korea University Guro Hospital, Korea University College of Medicine, Seoul, Korea

^17^Department of Surgery, Kyung Hee University Hospital at Gangdong, School of Medicine, Kyung Hee University, Seoul, Korea

^18^Department of Surgery, Ewha Womans University Mokdong Hospital, Ewha Womans University College of Medicine, Seoul, Korea

^19^Department of Medicine, Yonsei University College of Medicine, Seoul, Korea

^20^Division of Breast and Endocrine Surgery, Department of Surgery, Korea University Ansan Hospital, Korea University College of Medicine, Seoul, Korea

^21^Department of Surgery, Dankook University College of Medicine, Cheonan-si, Korea

^22^Department of Breast and Endocrine Surgery, Hallym University Sacred Heart Hospital, Hallym University, Anyang, Korea

^23^Department of Surgery, Incheon St. Mary’s Hospital, The Catholic University of Korea, Incheon, Korea

^24^Department of Surgery, Samsung Changwon Hospital, Sungkyunkwan University School of Medicine, Changwon, Korea

^25^Department of Surgery, University of Ulsan College of Medicine, Asan Medical Center, Seoul, Korea

* Hong-Kyu Kim and Dong Seung Shin have contributed equally to this work.

† Present address: Department of Surgery, Seoul National University Hospital, Seoul National University College of Medicine, Seoul, Korea

**Corresponding Author: Jai Min Ryu, MD, PhD**

Division of Breast Surgery, Department of Surgery, Samsung Medical Center, Sungkyunkwan University School of Medicine, 81 Irwon-ro, Gangnam-gu, Seoul, 06351, South Korea

Tel: +82-2-3410-3476, Fax: +82-2-3410-6982

E-mail: sheol1981@naver.com, jaimin.ryu@samsung.com

**&**

**Co-corresponding Author: Sae Byul Lee, MD, PhD**

Department of Surgery, University of Ulsan College of Medicine, Asan Medical Center, Seoul, Korea

E-mail: newstar153@hanmail.net

**Supplementary Materials - Index**

| **Supplementary Figures and Tables** |  |
| --- | --- |
| TABLE S1. Summary of BRCA1/2 mutation test results of the patients | *page 4* |
| Figure S1. Kaplan-Meier survival curves based on BRCA 1/2 status. | *page 5-6* |

**Supplementary Figures and Tables**

**Table S1.** **Summary of *BRCA1/2* mutation test results of the patients**

| ***BRCA1/2* result** | **Individuals tested for *BRCA1/2* mutation (*n* = 787)** | |
| --- | --- | --- |
|  | ***n*** | **(%)** |
| *BRCA1* PV/LPV | 85 | (10.8%) |
| *BRCA2* PV/LPV | 101 | (12.8%) |
| Negative | 522 | (66.3%) |
| Variant of unknown significance | 76 | (9.7%) |
| Equivocal | 3 | (0.4%) |

PV/LPV, pathologic variant/likely pathologic variant.

1. **Regional recurrence-free survival**


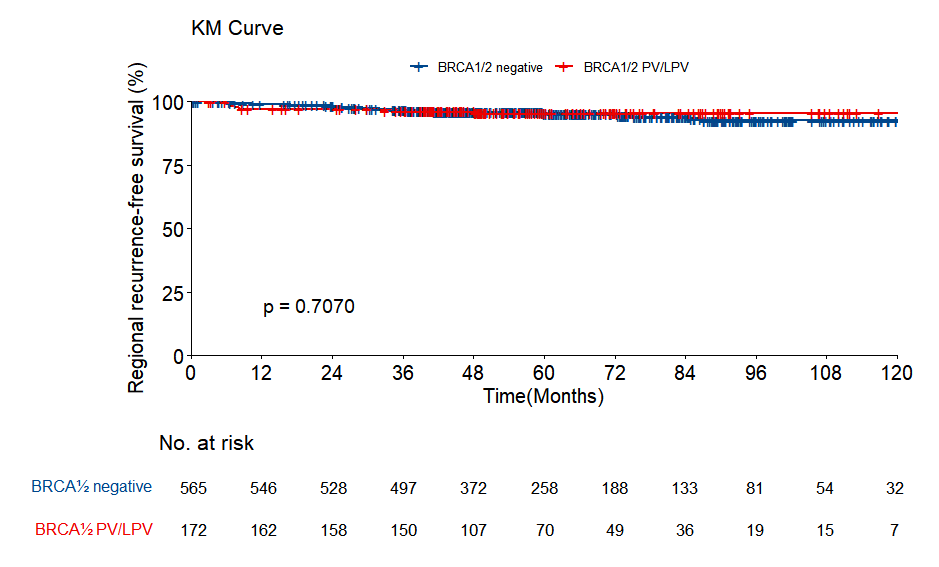


1. **Distant metastasis-free survival**


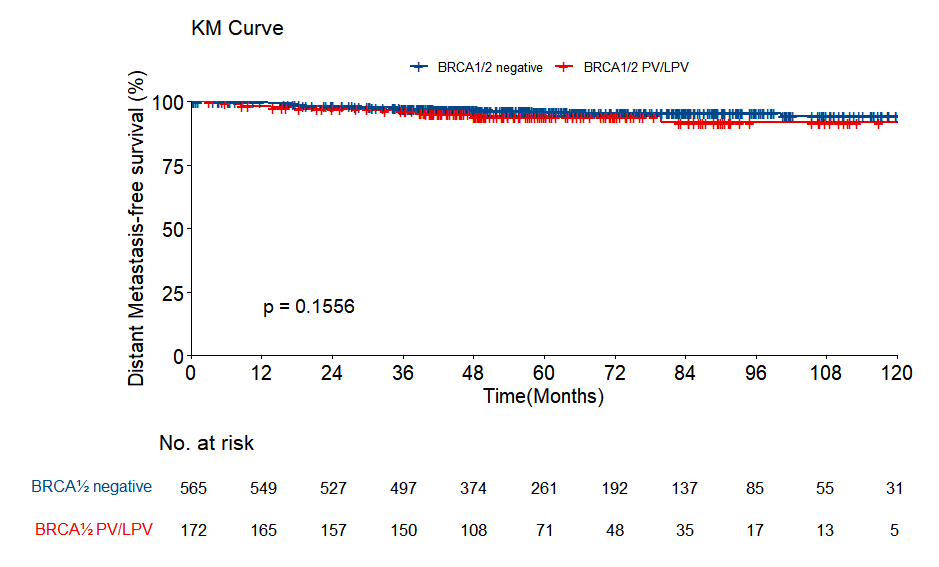


1. **Overall survival**


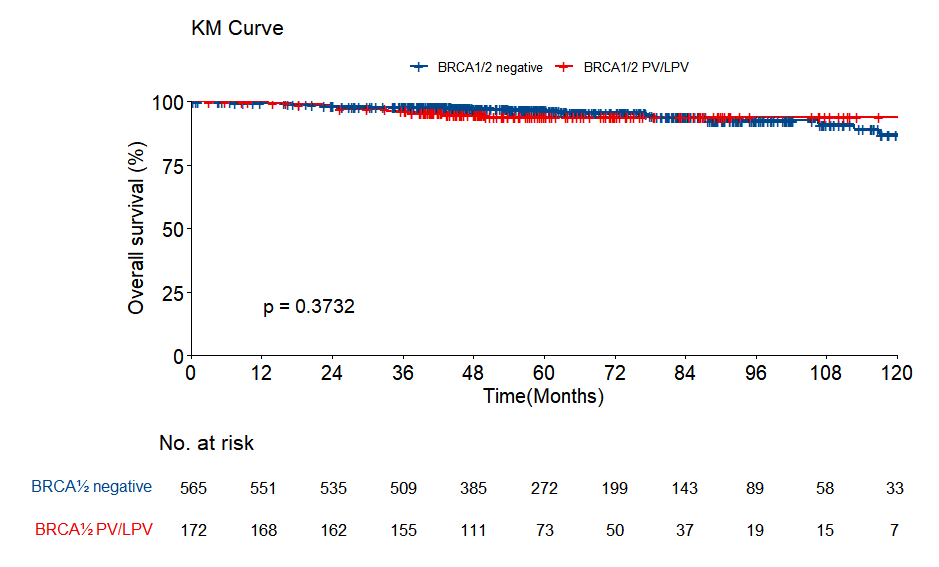


**Figure S1. Kaplan-Meier survival curves based on *BRCA 1/2* status.**

(A) Regional recurrence-free survival. (B) Distant metastasis-free survival. (C) Overall survival. In this analysis, we included 737 patients with unilateral breast cancer, excluding 9 patients who underwent unilateral total mastectomy, to evaluate the impact of NSM on ipsilateral local recurrence-free survival. *P* values are from the log-rank test.
